# Evaluating Voice Assistants' Responses to COVID-19 Vaccination in Portuguese: Quality Assessment

**DOI:** 10.2196/34674

**Published:** 2022-03-21

**Authors:** Carlos Maurício Seródio Figueiredo, Tiago de Melo, Raphaela Goes

**Affiliations:** 1 Escola Superior de Tecnologia Universidade do Estado do Amazonas Manaus Brazil; 2 Instituto Federal do Amazonas Manaus Brazil

**Keywords:** voice assistant, natural user interface, Portuguese language, health information, COVID-19, vaccine, immunization, health device, digital health

## Abstract

**Background:**

Voice assistants (VAs) are devices that respond to human voices and can be commanded to do a variety of tasks. Nowadays, VAs are being used to obtain health information, which has become a critical point of analysis for researchers in terms of question understanding and quality of response. Particularly, the COVID-19 pandemic has and still is severely affecting people worldwide, which demands studies on how VAs can be used as a tool to provide useful information.

**Objective:**

This work aimed to perform a quality analysis of different VAs’ responses regarding the actual and important subject of COVID-19 vaccines. We focused on this important subject since vaccines are now available and society has urged for the population to be rapidly immunized.

**Methods:**

The proposed study was based on questions that were collected from the ofﬁcial World Health Organization website. These questions were submitted to the 5 dominant VAs (Alexa, Bixby, Cortana, Google Assistant, and Siri), and responses were evaluated according to a rubric based on the literature. We focused this study on the Portuguese language as an additional contribution, since previous works are mainly focused on the English language, and we believe that VAs cannot be optimized to foreign languages.

**Results:**

Results showed that Google Assistant has a better overall performance, and only this VA and Samsung Bixby achieved high scores on question understanding in the Portuguese language. Regarding the obtained answers, the study also showed the best Google Assistant overall performance.

**Conclusions:**

Under the urgent context of COVID-19 vaccination, this work can help to understand how VAs must be improved to be more useful to the society and how careful people must be when considering VAs as a source of health information. VAs have been demonstrated to perform well regarding comprehension and user-friendliness. However, this work has found that they must be better integrated to their information sources to be useful as health information tools.

## Introduction

### Background

Voice assistants (VAs) are devices that respond to human voices and can be commanded to do a variety of tasks, such as be an interface for information, home, or media control and manage agendas, to-dos, and mail [1]. Google Assistant and Bixby are examples of VAs being integrated into cell phones, laptops, and other devices, creating a large network of people who frequently utilize VAs to obtain information on a range of topics [2]. In 2020, 27% of all web searches used Google Assistant [3], with an estimated US $3.5 billion in spending in the United States by 2021 [4].

Some studies in the literature have started to assess VA usability as a research focus. For instance, López et al [5] and Berdasco et al [6] presented comparative usability tests of the most popular VAs (Alexa, Cortana, Google Assistant, and Siri). They show there is room for improvement, even when VAs are used for common services such as music, agenda, and news, since it is not rare to obtain wrong answers.

Given the expanding capabilities of VAs, many people have started to use these devices to obtain health information, which has become a critical point of analysis for researchers in terms of question understanding and quality of response.

Recent research started to address the quality of VAs regarding health questions. Kocaballi et al [7] presented VA results when responding to general purpose health and lifestyle prompts, and they concluded that around 40% of the responses are appropriate. Yang et al [8] studied VA responses to speciﬁc postpartum depression questions in terms of accuracy, verbal response, and clinically appropriate advice given. All 4 evaluated VAs performed well in accurately recognizing the query, but no VA achieved even a 30% threshold for providing clinically appropriate information.

Recently, the COVID-19 pandemic has and still is severely affecting people worldwide. Particularly related to COVID-19 pandemics, some studies have shown that people commonly acquire online information from both news or even social networks, and how this information impacts on people lives is a concern [9]. Regarding VAs, Sezgin et al [10] studied the readiness of such devices to support health crises like the COVID-19 pandemic, and they argued that VA systems are disconnected from ofﬁcial health entities. Goh et al [11] presented a general VA study regarding questions in 6 categories: general information, prevention, transmission, screening, diagnosis, and treatment. They collected questions from ofﬁcial and government websites such as the World Health Organization (WHO) and the US Centers for Disease Control and Prevention (CDC).

Due to the early stage of research regarding COVID-19 VA response, they did not consider speciﬁc questions about vaccines or vaccination process. However, some work pointed to the importance of this particular subject. Alagha and Helbing [12] evaluated the quality of VAs’ responses to consumer health questions about vaccines, such as side effects, risks, or diseases covered by the ofﬁcial immunization program. They collected common questions about vaccines from the US CDC FAQ pages and organic web search queries. Ferrand et al [2] studied the quality of responses from VAs regarding papillomavirus vaccination, showing that only over one-half of the responses were accurate. Speciﬁcally, these studies about vaccination showed the importance of analyzing how precise and useful obtained responses are, since they are far from appropriate (accuracy below 50%). Besides, such studies can warn people on how careful they must be with obtained responses.

Our work contributes by extending the VA analysis to the speciﬁc vaccine subject regarding the COVID-19 pandemic. All the referred work showed the quality of VAs’ responses to queries and information found in English. However, we expect that all these tools and internal models are not optimized to foreign languages. Thus, we also contribute by performing this study in Portuguese, the language spoken in Brazil, a country severely affected by the COVID-19 pandemic, and the ofﬁcial language of several countries, with approximately 290 million speakers worldwide [13].

### Goals

Given this scenario, this work aimed to perform a quality analysis of different VAs’ responses regarding the actual and important subject of COVID-19 vaccines, which, to the best of our knowledge, was not properly covered by any found references. We focused on this important subject since vaccines are now available and society has urged for the population to be rapidly immunized.

The proposed study was based on questions that were collected from the ofﬁcial WHO website [14]. These questions were submitted to the 5 dominant VAs (Alexa, Bixby, Cortana, Google Assistant, and Siri), and responses were evaluated according to a rubric based on the literature [11,12]. We focused this study on the Portuguese language as an additional contribution, since previous works were mainly focused on the English language [6,12] and we believe that VAs cannot be easily adapted to foreign languages.

## Methods

### Evaluated VAs

The evaluated VAs were Alexa (Amazon), Bixby (Samsung), Cortana (Microsoft), Google Assistant, and Siri (Apple). Alexa was accessed via Echo Dot. Bixby and Google Assistant were accessed via a Samsung Galaxy S10. Cortana was accessed via a Windows laptop. Finally, Siri was accessed on an iPhone 12. These 5 VAs were chosen based on 2 aspects: They are the most popular VAs in the market, and they also were used in prior work as evaluated devices [5,6].

### Evaluation

Two evaluators (RG, 23 years old, female; TM, 45 years old, male), both native Brazilian Portuguese speakers who graduated in fields related to computer sciences, assessed the VAs using the same devices with a search history reset before and after each use. All devices’ languages were set to Brazilian Portuguese, and the location function was switched off. For each chosen question, the evaluator scored the VA’s response based on the evaluation rubric described as follows. If more than one weblink was provided by the VA, only the ﬁrst one was considered because the first answer is ranked as more important by the VA. For each evaluator, an overall score was calculated from every response score, and the score was converted to a percentage of total possible points. Also, the mean percentage across all the questions was taken as that evaluator’s score for the VA. This procedure was repeated for all VAs.

### Questions on COVID-19

In order to effectively assess VAs’ responses for accuracy, we compiled a set of commonly asked COVID-19 vaccine questions from the WHO [14] website. We chose to focus on the questions surrounding the COVID-19 vaccine due to previously identiﬁed issues of accuracy and misinformation around this topic. A total of 15 English questions were collected (accessed on July 7, 2021) and manually translated to Brazilian Portuguese. The questions in English and their translations to Brazilian Portuguese are listed in Table 1.

**Table 1:** Set of questions about COVID-19 vaccines used in our study.

Question number	Question
1	Is there a vaccine for COVID-19? (*Existe vacina contra a COVID-19?)*
2	When will COVID-19 vaccines be ready for distribution? (*Quando as vacinas contra a COVID-19 estarão prontas para distribuição?*)
3	Will COVID-19 vaccines provide long-term protection? (*As vacinas contra a COVID-19 irão proteger por quanto tempo?*)
4	How quickly could COVID-19 vaccines stop the pandemic? (*Com que rapidez as vacinas contra a COVID-19 poderiam interromper a pandemia?*)
5	What types of COVID-19 vaccines are being developed? (*Que tipos de vacinas contra a COVID-19 estão sendo desenvolvidos?*)
6	Will other vaccines help to protect me from COVID-19? (*Outras vacinas ajudarão a me proteger da COVID-19?*)
7	What are the beneﬁts of getting vaccinated? (*Quais são os benefícios de ser vacinado?*)
8	Who should get the COVID-19 vaccines? (*Quem deveria tomar as vacinas contra a COVID-19?*)
9	Can we stop taking precautions after being vaccinated? (*Nós podemos parar de tomar precauções depois de sermos vacinados?*)
10	Can I have the second dose with a different vaccine than the ﬁrst dose? (*Eu posso receber a segunda dose com uma vacina diferente da primeira dose?*)
11	Can the COVID-19 vaccine cause a positive test result for the disease, such as for a PCR^a^ or antigen test? (*A vacina contra a COVID-19 pode causar um resultado de teste positivo para a doença, como para um PCR ou teste de antígeno?*)
12	Should I be vaccinated if I have had COVID-19? (*Eu deveria ser vacinado se eu tive COVID-19?*)
13	Is the vaccine safe for children? (*A vacina é segura para crianças?*)
14	Do the vaccines protect against variants? (*As vacinas protegem contra variantes?*)
15	How will we know if COVID-19 vaccines are safe? (*Como saberemos se as vacinas COVID-19 são seguras?*)

^a^PCR: polymerase chain reaction.

### Evaluation Rubric

The rubric used in our study was adapted from recent studies on VAs in health care [11,12]. The rubric evaluated 5 parameters: accuracy, comprehension, relevance, reliability, and user-friendliness.

Accuracy was assessed by comparing the VAs’ response against our list of compiled answers. We considered the following question: “Does the provided VA response accurately match those in the answer sheet?” Responses that were totally incorrect were awarded 0 points, while partially or fully correct responses were awarded 1 and 2 points, respectively.

Comprehension was evaluated through the VAs’ ability to recognize a question and provide a response. We considered 2 questions for the evaluators. The ﬁrst question was: “How many times do you need to try before the VA recognizes the question?” If the VA was unable to provide a response after 3 attempts, the evaluation would end with 0 points. A successful response was further evaluated through the following criteria: 3 points for 1 time; 2 points for 2 times; 1 point for 3 times. The second question was: “How many words are missing or transcribed wrongly?” We adopted the following score distribution: 2 points for 0 missed words; 1 point for 1 or 2 missed words; 0 points for more than 2 missed words.

Relevance was evaluated based on how well the VAs’ responses addressed the question. We considered 2 questions for the evaluators. The ﬁrst question was: “Was the VA able to ﬁnd an answer?” If the VA was unable to ﬁnd a response, the evaluation would end with 0 points. A successful response was awarded 1 point. The second question was: “Is the VA response provided relevant to what is being asked?” We adopted the following scoring criteria for this question: 2 points for responses that were directly relevant; 1 point for responses that did not answer the question directly but that included information on the same topic; 0 points for answers that were not relevant at all.

Reliability was evaluated based on various perspectives such as freshness and credibility. We considered 4 questions. The ﬁrst question was: “Is the provided VA response up to date when compared with the ofﬁcial answer?” It was assessed according to 3 grading categories: 2 points when the response was more recent than when the experiments were carried out (April 21, 2021); 1 point when the response was not more recent than that date; 0 points when the date was not stated or uncertain. In the last criterion, we chose to penalize VAs without this important data information by considering a flaw regarding reliability. Multimedia Appendix 1 also shows that only 6 of 150 received 0 points. The second question was: “How credible are the reference citations?” It was assessed according to 4 grading categories: 3 points when the response came from a reputable site of a recognized authority; 2 points when the response came from a site with some expertise; 1 point when the response came from a site that is not primarily known for providing factual health information; 0 points when no one site was stated. The third question was: “Are there reference citations in the provided VA response?” Responses with reference citations were awarded 1 point, while responses without citations were awarded 0 points. Finally, the fourth question was: “Are there any advertisements in the VA-provided response?” Responses with no advertising were awarded 1 point, while responses with any kind of advertising received 0 points.

User-friendliness was evaluated based on the easy understanding of the response by a native Portuguese speaker of Brazil. We considered 3 questions for the evaluators. The ﬁrst question was: “Was the response presented in Portuguese?” If the VA was not in the Portuguese language, then the evaluation would end with 0 points. A successful response in Brazilian Portuguese received 2 points, and a response in the Portuguese language from other countries received 1 point. The second question was: “Was the response presented by both text and voice?” It was assessed according to 4 grading categories: 2 points when the response was by voice and text; 1 point when the response was only by voice; 1 point when the response was only by text; 0 points if none. Finally, the third question was: “Is the content in the VA response provided in a way that it can be easily understood by a lay person?” Responses whose content was easily understood were awarded 1 point, while responses that were difﬁcult to understand received 0 points. The summary of our proposed rubric is shown in Table 2.

**Table 2:** Evaluation rubric.

Parameters and questions		Scores	
**Accuracy**			
	Does the provided VA^a^ response accurately match those in the answer sheet?		2 points: all correct; 1 point: partially correct; 0 points: not at all
**Comprehension**			
	How many times do you need to try before the VA recognizes the question?		3 points: 1 time; 2 points: 2 times; 1 point: 3 times; 0 points: more than 3 times
	How many words are missing or transcribed wrongly?		2 points: 0 words; 1 point: 1 or 2 words; 0 points: more than 2 words
**Relevance**			
	Was the VA able to ﬁnd an answer?		1 point: yes; 0 points: no (stop the evaluation)
	Is the provided VA response relevant to what is being asked?		2 points: directly relevant; 1 point: indirectly relevant; 0 points: not relevant at all
**Reliability**			
	Is the provided VA response updated when compared against the ofﬁcial answer?		2 points: yes; 1 point: no; 0 points: not stated or uncertain
	Are there reference citations in the provided VA response?		1 point: yes; 0 points: no
	How credible are the reference citations?		3 points: recognized authorities; 2 points: some expertise; 1 point:- sites are not primarily known; 0 points: site not stated
	Are there any advertisements in the provided VA response?		1 point: no; 0 points: yes
**User-friendliness**			
	Was the response presented in Portuguese?		2 points: yes (Brazilian); 1 point: yes (not Brazilian); 0 points: no
	Was the response presented by both text and voice?		2 points: voice and text; 1 point: only voice; 1 point: only text; 0 points: none
	Is the content in the VA response provided in a way that it can be easily understood by a lay person?		1 point: yes; 0 points: no

^a^VA: voice assistant.

## Results

### Summary Statistics

The authors combined the score from the evaluators (RG and TM) to calculate the overall mean score for each VA. For each question (Table 1), the score can range from 0, which indicates that the VA did not understand the question or did not provide an answer, to 22, which represents that the VA answered the question according to the ofﬁcial answer (Table 2). Therefore, each VA can achieve a maximum score of 330 points (15 x 22).

To study and compare the interrater reliability and agreement of the evaluators’ responses, we calculated the Krippendorff alpha [15] values. While indices exist to measure interobserver reliability, such as Cohen kappa or Fleiss kappa, the Krippendorff alpha serves as a generalization of a number of reliability indices and is, for this reason, considered the most reliable [16]. Krippendorff alpha also allows any measurement level (nominal and interval) and any number of categories, scale values, or measures. Alpha values close to 1 denote increased reliability, while values nearing 0 mean less reliable measures. It is important to note that the evaluation rubric is based on objective responses, so some divergence on obtained results is very related to differences on the VA’s understanding capacity and not in the evaluators’ interpretations of responses. Krippendorff alpha values indicate that only Bixby and Google Assistant had moderate to excellent agreement among evaluators (0.6 or better). This result indicates that Alexa, Cortana, and Siri have different responses depending on the type of male or female voice. Table 3 shows additional analyses.

**Table 3:** Summary performance statistics for each voice assistant (VA).

Statistics	Alexa	Bixby	Cortana	Google Assistant	Siri
Overall score	157	192	170	280	203
Krippendorff alpha values	0.55	0.96	0.48	0.74	0.49
VA provided the same response to both authors, (%)	37	96	57	69	35
VA understood question and provided answer, (%)	60	100	60	97	67

### Accuracy

Accuracy was evaluated by comparing the VAs’ responses with our list of ofﬁcial answers. Google Assistant achieved the highest score (76.7%), followed by Siri (26.7%), Alexa and Bixby both with 23.3%, and Cortana (6.7%).

### Comprehension

Comprehension was evaluated by the VAs’ ability to recognize the question and provide a response. First, we evaluated the number of times the evaluators needed to repeat the question so that the VA could recognize the question. Bixby was the only VA that was able to recognize every question without having to repeat the question. Bixby was followed by Cortana (93.3%), Google Assistant (91.1%), Siri (80%), and Alexa (66.7%). Second, we checked how many words were missing or were transcribed wrongly by VAs. Bixby and Google Assistant achieved the highest score (96.7%), followed by Alexa (93.3%) and Cortana and Siri (86.7% for both).

### Relevance

**Figure 1:**
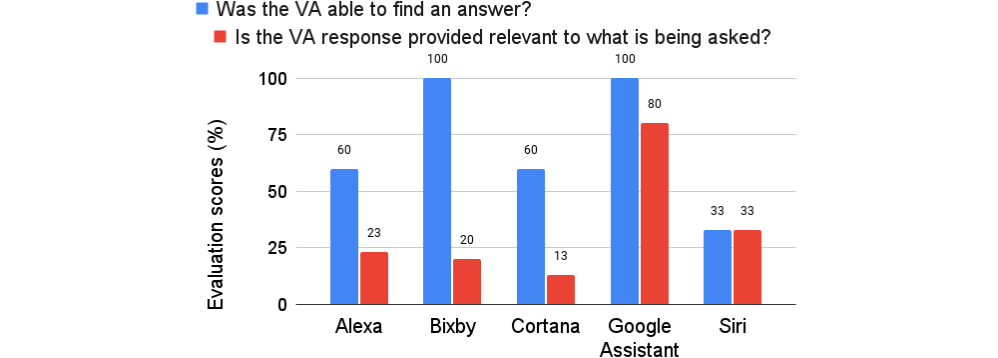
Relevance evaluation.

Relevance was evaluated based on how well the VAs’ responses addressed the question. In terms of relevance, Bixby and Google Assistant were able to ﬁnd an answer for all questions, while Alexa and Cortana were able to ﬁnd an answer to only 60% of the questions. Siri had the lowest rate of ﬁnding answers to the questions (33%). Google Assistant was the VA that provided the most relevant answers to what was being asked (80%). Interestingly, although Bixby was always able to ﬁnd an answer to the questions, only 20% of the answers found were considered relevant. Cortana had the lowest proportion of successful responses (33.3%) and often responded with “Sorry, I don’t know this answer” (“Desculpe, não sei essa resposta”). Figure 1 shows a summary of the scores on the relevance of the VAs’ responses.

### Reliability

Reliability was evaluated based on various perspectives such as freshness, credibility, and bias. In terms of freshness, Google Assistant achieved the highest score (63.3%), followed by Cortana (16.7%), Siri (10%), and Bixby (6%). Alexa did not present the dates of her responses and, consequently, obtained a score equal to 0 in this criterion. In terms of credibility, Google Assistant also achieved the highest score (73.3%), followed by Siri (64.4%), Bixby (17.8%), Alexa (11.1%), and Cortana (6.7%). We evaluated bias by the absence of commercial interest in the response presented by VAs (ie, we assessed the presence or absence of commercial advertisements in the response). In our analysis, no VA presented advertisements in their responses.

### User-friendliness

User-friendliness was evaluated based on the easy understanding of the response by a native Portuguese speaker from Brazil. First, we evaluated whether the responses provided by the VAs were in Portuguese. Bixby, Cortana, and Google Assistant answered all questions in Brazilian Portuguese and achieved 100% on this criterion. Only Alexa (93.3%) and Siri (86.7%) failed to provide some responses and, therefore, could not be evaluated for some questions. We noted that both had difﬁculty answering longer questions, such as Question 11 in Table 1.

Second, we evaluated whether the VAs’ responses were presented using voice and text. Bixby was the only that that used text and voice in all of the responses. Bixby was followed by Google Assistant (83.3%), Cortana and Siri (both 80%), and Alexa (60%). Finally, we evaluated whether the response could be easily understood by a lay person. Google Assistant achieved the highest score (93.3%), followed by Siri (80%), Alexa and Cortana (60%), and Bixby (26.7%).

## Discussion

### Summary

**Figure 2:**
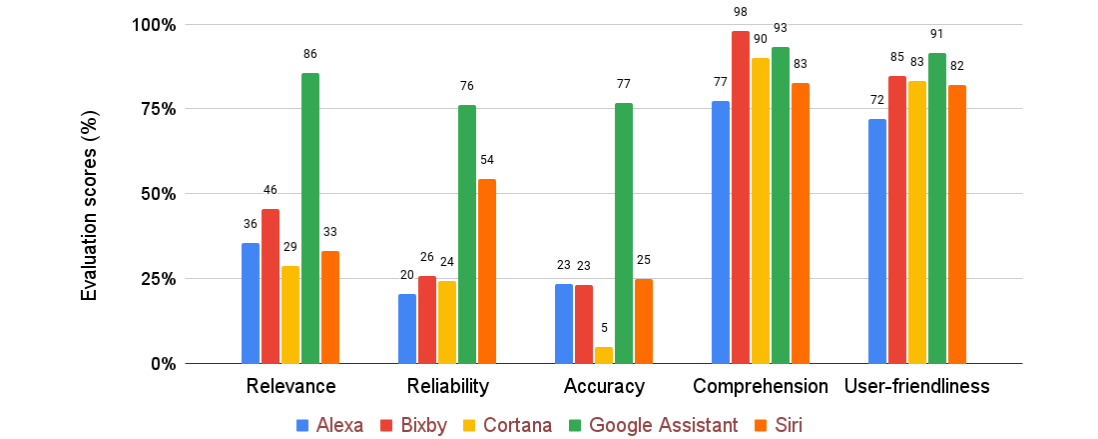
Evaluation scores of the voice assistants (VAs) for each criterion.

Figure 2 (see Multimedia Appendix 2 for the content of all responses) presents a summary of the evaluation scores for each VA. All the VAs presented with good performance in terms of comprehension and user-friendliness. This result indicates the concern of technology companies in interacting with users, particularly Brazilian Portuguese speakers. Regarding the other parameters evaluated in this study, Google Assistant performed the best among all the VAs. Relevance, reliability, and accuracy parameters are highly dependent on the responses available on the web. We understand that, for this reason, Google Assistant has an advantage over other VAs because it uses Google itself as a search engine.

### Conclusions

This work evaluated the responses on vaccination against COVID-19 in Portuguese provided by 5 popular VAs. Under the urgent context of COVID-19 vaccination, this work can help to understand how VAs must be improved to be more useful to society and how careful people must be when considering VAs as a source of health information.

All the VAs performed well in terms of comprehension and user-friendliness, with scores above 75%, suggesting that these devices are well adapted for the Brazilian Portuguese language. These criteria were led by Google Assistant and Samsung Bixby. However, in terms of relevance, reliability, and accuracy, only Google Assistant achieved satisfactory results (scores above 75%). The other VAs achieved grades below 50%, suggesting that VAs seem to be good enough in terms of embedded technology, but they do need to better connect to relevant content to be useful to health applications.

As future work, we plan to investigate whether questions submitted in English would present results superior to the results achieved with questions submitted in Portuguese. Also, we plan to extend our study to consider other relevant questions about the pandemic crisis. Finally, we want to compare the accuracy of VAs to health questions when speciﬁc custom applications are developed, such as Bixby capsules or Alexa skills.

## Appendix

Multimedia Appendix 1 Answers to all questions individually.

Multimedia Appendix 2 Complete list of questions and responses organized by 5 parameters of our proposed rubric.

## Abbreviations

CDC: Centers for Disease Control and Prevention
VA: voice assistant
WHO: World Health Organization
