# Supplementary material for: Evaluating Voice Assistants' Responses to COVID-19 Vaccination in Portuguese: Quality Assessment
Source: JMIR Hum Factors. 2022 Mar 21;9(1):e34674. doi: 10.2196/34674 (PMC8942094; doi:10.2196/34674)
Supplement: Multimedia Appendix 1 [file humanfactors_v9i1e34674_app1.pdf]

APPENDIX 1

the answers to all questions individually

ALEXA

#Q	Question	RG (female)					TM (male)				
1	Is there a vaccine for COVID-19?<br>*(Existe vacina contra a COVID-19?)*	3	2	2	5	5	3	5	2	5	5
2	When will COVID-19 vaccines be ready for distribution?<br>*(Quando as vacinas contra a COVID-19 estarão prontas para distribuição?)*	1	1	0	3	3	0	1	0	5	4
3	Will COVID-19 vaccines provide long-term protection?<br>*(As vacinas contra a COVID-19 irão proteger por quanto tempo?)*	3	2	2	5	5	3	5	2	5	5
4	How quickly could COVID-19 vaccines stop the pandemic?<br>*(Com que rapidez as vacinas contra a COVID-19 poderiam interromper a pandemia?)*	1	1	0	3	3	0	1	0	5	4
5	What types of COVID-19 vaccines are being developed?<br>*(Que tipos de vacinas contra a COVID-19 estão sendo desenvolvidos?)*	3	2	2	4	5	0	1	0	5	4
6	Will other vaccines help to protect me from COVID-19?<br>*(Outras vacinas ajudarão a me proteger da COVID-19?)*	1	1	0	3	3	0	1	0	5	4
7	What are the benefits of getting vaccinated?<br>*(Quais são os benefícios de ser vacinado?)*	1	1	0	3	3	0	1	0	5	4
8	Who should get the COVID-19 vaccines?<br>*(Quem deveria tomar as vacinas contra a COVID-19?)*	1	1	0	3	3	0	1	0	5	4
9	Can we stop taking precautions after being vaccinated?<br>*(Nós podemos parar de tomar precauções depois de sermos vacinados?)*	1	1	0	3	3	0	0	0	0	0
10	Can I have the second dose with a different vaccine than the first dose?<br>*(Eu posso receber a segunda dose com uma vacina diferente da primeira dose?)*	1	1	0	3	3	0	1	0	5	4
11	Can the COVID-19 vaccine cause a positive test result for the disease, such as for a PCR or antigen test?<br>*(A vacina contra a COVID-19 pode causar um resultado de teste positivo para a doença, como para um PCR ou teste de antígeno?)*	1	1	0	3	3	0	0	0	0	0
12	Should I be vaccinated if I have had COVID-19?<br>*(Eu deveria ser vacinado se eu tive COVID-19?)*	1	1	0	3	3	0	1	0	5	4
13	Is the vaccine safe for children?<br>*(A vacina é segura para crianças?)*	1	1	0	3	3	0	1	0	5	4
14	Do the vaccines protect against variants?<br>*(As vacinas protegem contra variantes?)*	3	1	2	3	4	0	1	0	5	4
15	How will we know if COVID-19 vaccines are safe?<br>*(Como saberemos se as vacinas COVID-19 são seguras?)*	1	1	0	4	4	3	5	2	5	5

Relevance
Reliability
Accuracy
Comprehension
User-friendliness

BIXBY

#Q	Question	RG (female)					TM (male)				
1	Is there a vaccine for COVID-19?<br>*(Existe vacina contra a COVID-19?)*	2	1	1	5	5	2	1	1	5	5
2	When will COVID-19 vaccines be ready for distribution?<br>*(Quando as vacinas contra a COVID-19 estarão prontas para distribuição?)*	1	1	0	5	4	1	1	0	5	4
3	Will COVID-19 vaccines provide long-term protection?<br>*(As vacinas contra a COVID-19 irão proteger por quanto tempo?)*	1	1	0	5	4	1	1	0	5	4
4	How quickly could COVID-19 vaccines stop the pandemic?<br>*(Com que rapidez as vacinas contra a COVID-19 poderiam interromper a pandemia?)*	1	1	0	5	4	1	1	0	5	4
5	What types of COVID-19 vaccines are being developed?<br>*(Que tipos de vacinas contra a COVID-19 estão sendo desenvolvidos?)*	2	5	2	5	5	2	5	2	5	5
6	Will other vaccines help to protect me from COVID-19?<br>*(Outras vacinas ajudarão a me proteger da COVID-19?)*	1	1	0	5	4	1	1	0	5	4
7	What are the benefits of getting vaccinated?<br>*(Quais são os benefícios de ser vacinado?)*	3	5	2	5	5	3	5	2	5	5
8	Who should get the COVID-19 vaccines?<br>*(Quem deveria tomar as vacinas contra a COVID-19?)*	1	1	0	5	4	1	1	0	5	4
9	Can we stop taking precautions after being vaccinated?<br>*(Nós podemos parar de tomar precauções depois de sermos vacinados?)*	1	1	0	5	4	1	1	0	5	4
10	Can I have the second dose with a different vaccine than the first dose?<br>*(Eu posso receber a segunda dose com uma vacina diferente da primeira dose?)*	1	1	0	4	4	1	1	0	5	4
11	Can the COVID-19 vaccine cause a positive test result for the disease, such as for a PCR or antigen test?<br>*(A vacina contra a COVID-19 pode causar um resultado de teste positivo para a doença, como para um PCR ou teste de antígeno?)*	1	1	0	5	4	1	1	0	5	4
12	Should I be vaccinated if I have had COVID-19?<br>*(Eu deveria ser vacinado se eu tive COVID-19?)*	1	1	0	4	4	1	1	0	4	4
13	Is the vaccine safe for children?<br>*(A vacina é segura para crianças?)*	1	1	0	5	4	1	1	0	5	4
14	Do the vaccines protect against variants?<br>*(As vacinas protegem contra variantes?)*	3	5	2	5	5	2	5	2	5	4
15	How will we know if COVID-19 vaccines are safe?<br>*(Como saberemos se as vacinas COVID-19 são seguras?)*	1	1	0	5	4	1	1	0	5	4

Relevance
Reliability
Accuracy
Comprehension
User-friendliness

CORTANA

#Q	Question	RG (female)					TM (male)				
1	Is there a vaccine for COVID-19?<br>*(Existe vacina contra a COVID-19?)*	2	6	0	5	5	0	1	0	4	4
2	When will COVID-19 vaccines be ready for distribution?<br>*(Quando as vacinas contra a COVID-19 estarão prontas para distribuição?)*	1	1	0	2	4	2	5	1	5	5
3	Will COVID-19 vaccines provide long-term protection?<br>*(As vacinas contra a COVID-19 irão proteger por quanto tempo?)*	1	1	0	5	4	0	1	0	4	4
4	How quickly could COVID-19 vaccines stop the pandemic?<br>*(Com que rapidez as vacinas contra a COVID-19 poderiam interromper a pandemia?)*	1	1	0	5	4	0	1	0	4	4
5	What types of COVID-19 vaccines are being developed?<br>*(Que tipos de vacinas contra a COVID-19 estão sendo desenvolvidos?)*	1	1	0	5	4	0	1	0	4	4
6	Will other vaccines help to protect me from COVID-19?<br>*(Outras vacinas ajudarão a me proteger da COVID-19?)*	1	1	0	5	4	0	1	0	5	4
7	What are the benefits of getting vaccinated?<br>*(Quais são os benefícios de ser vacinado?)*	1	1	0	5	4	0	1	0	5	4
8	Who should get the COVID-19 vaccines?<br>*(Quem deveria tomar as vacinas contra a COVID-19?)*	1	1	0	2	4	0	1	0	5	4
9	Can we stop taking precautions after being vaccinated?<br>*(Nós podemos parar de tomar precauções depois de sermos vacinados?)*	1	1	0	5	4	0	1	0	4	4
10	Can I have the second dose with a different vaccine than the first dose?<br>*(Eu posso receber a segunda dose com uma vacina diferente da primeira dose?)*	1	1	0	5	4	0	1	0	5	4
11	Can the COVID-19 vaccine cause a positive test result for the disease, such as for a PCR or antigen test?<br>*(A vacina contra a COVID-19 pode causar um resultado de teste positivo para a doença, como para um PCR ou teste de antígeno?)*	1	1	0	5	4	0	1	0	5	4
12	Should I be vaccinated if I have had COVID-19?<br>*(Eu deveria ser vacinado se eu tive COVID-19?)*	1	1	0	5	4	0	1	0	4	4
13	Is the vaccine safe for children?<br>*(A vacina é segura para crianças?)*	1	1	0	5	4	3	5	1	5	5
14	Do the vaccines protect against variants?<br>*(As vacinas protegem contra variantes?)*	3	5	0	4	5	3	5	1	5	5
15	How will we know if COVID-19 vaccines are safe?<br>*(Como saberemos se as vacinas COVID-19 são seguras?)*	1	1	0	4	4	0	1	0	4	4

Relevance
Reliability
Accuracy
Comprehension
User-friendliness

GOOGLE ASSISTANT

#Q	Question	RG (female)					TM (male)				
1	Is there a vaccine for COVID-19?<br>*(Existe vacina contra a COVID-19?)*	3	7	2	5	5	3	7	2	5	5
2	When will COVID-19 vaccines be ready for distribution?<br>*(Quando as vacinas contra a COVID-19 estarão prontas para distribuição?)*	3	6	1	5	5	3	6	1	5	5
3	Will COVID-19 vaccines provide long-term protection?<br>*(As vacinas contra a COVID-19 irão proteger por quanto tempo?)*	2	7	0	5	5	3	4	2	5	4
4	How quickly could COVID-19 vaccines stop the pandemic?<br>*(Com que rapidez as vacinas contra a COVID-19 poderiam interromper a pandemia?)*	3	5	2	5	4	3	5	2	5	4
5	What types of COVID-19 vaccines are being developed?<br>*(Que tipos de vacinas contra a COVID-19 estão sendo desenvolvidos?)*	2	5	0	4	5	3	5	2	4	5
6	Will other vaccines help to protect me from COVID-19?<br>*(Outras vacinas ajudarão a me proteger da COVID-19?)*	3	5	2	5	4	3	5	2	5	4
7	What are the benefits of getting vaccinated?<br>*(Quais são os benefícios de ser vacinado?)*	3	7	2	5	5	3	7	2	5	5
8	Who should get the COVID-19 vaccines?<br>*(Quem deveria tomar as vacinas contra a COVID-19?)*	1	5	1	3	5	2	6	1	5	5
9	Can we stop taking precautions after being vaccinated?<br>*(Nós podemos parar de tomar precauções depois de sermos vacinados?)*	1	1	0	3	4	1	1	0	5	4
10	Can I have the second dose with a different vaccine than the first dose?<br>*(Eu posso receber a segunda dose com uma vacina diferente da primeira dose?)*	3	5	2	5	4	3	6	2	5	4
11	Can the COVID-19 vaccine cause a positive test result for the disease, such as for a PCR or antigen test?<br>*(A vacina contra a COVID-19 pode causar um resultado de teste positivo para a doença, como para um PCR ou teste de antígeno?)*	3	7	2	3	5	3	7	2	5	4
12	Should I be vaccinated if I have had COVID-19?<br>*(Eu deveria ser vacinado se eu tive COVID-19?)*	3	6	2	4	5	3	7	2	5	5
13	Is the vaccine safe for children?<br>*(A vacina é segura para crianças?)*	3	5	2	4	5	3	6	2	5	4
14	Do the vaccines protect against variants?<br>*(As vacinas protegem contra variantes?)*	2	6	2	5	5	0	1	0	5	4
15	How will we know if COVID-19 vaccines are safe?<br>*(Como saberemos se as vacinas COVID-19 são seguras?)*	3	5	2	5	5	3	5	2	5	4

Relevance
Reliability
Accuracy
Comprehension
User-friendliness

SIRI

#Q	Question	RG (female)					TM (male)				
1	Is there a vaccine for COVID-19?<br>*(Existe vacina contra a COVID-19?)*	1	4	0	5	5	3	4	2	5	4
2	When will COVID-19 vaccines be ready for distribution?<br>*(Quando as vacinas contra a COVID-19 estarão prontas para distribuição?)*	1	5	1	5	5	0	5	0	5	5
3	Will COVID-19 vaccines provide long-term protection?<br>*(As vacinas contra a COVID-19 irão proteger por quanto tempo?)*	1	5	1	5	5	3	5	2	3	4
4	How quickly could COVID-19 vaccines stop the pandemic?<br>*(Com que rapidez as vacinas contra a COVID-19 poderiam interromper a pandemia?)*	1	5	1	5	5	0	5	0	4	5
5	What types of COVID-19 vaccines are being developed?<br>*(Que tipos de vacinas contra a COVID-19 estão sendo desenvolvidos?)*	1	4	0	5	5	0	5	0	5	5
6	Will other vaccines help to protect me from COVID-19?<br>*(Outras vacinas ajudarão a me proteger da COVID-19?)*	1	4	0	5	5	0	5	0	5	5
7	What are the benefits of getting vaccinated?<br>*(Quais são os benefícios de ser vacinado?)*	1	2	0	5	5	3	5	2	5	4
8	Who should get the COVID-19 vaccines?<br>*(Quem deveria tomar as vacinas contra a COVID-19?)*	1	5	1	5	5	0	5	0	5	5
9	Can we stop taking precautions after being vaccinated?<br>*(Nós podemos parar de tomar precauções depois de sermos vacinados?)*	1	1	0	4	4	0	0	0	0	0
10	Can I have the second dose with a different vaccine than the first dose?<br>*(Eu posso receber a segunda dose com uma vacina diferente da primeira dose?)*	1	1	0	4	4	0	0	0	0	0
11	Can the COVID-19 vaccine cause a positive test result for the disease, such as for a PCR or antigen test?<br>*(A vacina contra a COVID-19 pode causar um resultado de teste positivo para a doença, como para um PCR ou teste de antígeno?)*	1	5	1	5	5	0	5	0	5	5
12	Should I be vaccinated if I have had COVID-19?<br>*(Eu deveria ser vacinado se eu tive COVID-19?)*	1	5	1	4	5	0	0	0	0	0
13	Is the vaccine safe for children?<br>*(A vacina é segura para crianças?)*	1	4	0	5	5	0	0	0	0	0
14	Do the vaccines protect against variants?<br>*(As vacinas protegem contra variantes?)*	1	4	0	5	5	3	6	2	5	4
15	How will we know if COVID-19 vaccines are safe?<br>*(Como saberemos se as vacinas COVID-19 são seguras?)*	1	4	0	5	5	3	6	1	5	4

Relevance
Reliability
Accuracy
Comprehension
User-friendliness
