# Supplementary material for: Evaluating Voice Assistants' Responses to COVID-19 Vaccination in Portuguese: Quality Assessment
Source: JMIR Hum Factors. 2022 Mar 21;9(1):e34674. doi: 10.2196/34674 (PMC8942094; doi:10.2196/34674)
Supplement: Multimedia Appendix 2 [file humanfactors_v9i1e34674_app2.pdf]

APPENDIX 2

Questions and Answers of Evaluation Rubric

ACCURACY

#Q	Question (accuracy)	Does the VA response provided accurately match those in the answer sheet?				
		Alexa	Bixby	Cortana	GA	Siri
1	Is there a vaccine for COVID-19?	1,5	0	1	**3**	2
2	When will COVID-19 vaccines be ready for distribution?	0	0	0,5	**3**	3
3	Will COVID-19 vaccines provide long-term protection?	1,5	0	0	2	**2,5**
4	How quickly could COVID-19 vaccines stop the pandemic?	0	0	0	2	**3**
5	What types of COVID-19 vaccines are being developed?	0	**3**	0	**3**	2,5
6	Will other vaccines help to protect me from COVID-19?	0	0	0	2	**2,5**
7	What are the benefits of getting vaccinated?	0	**3**	0	**3**	1
8	Who should get the COVID-19 vaccines?	0	0	0	2,5	**3**
9	Can we stop taking precautions after being vaccinated?	0	0	0	0	0
10	Can I have the second dose with a different vaccine than the first dose?	0	0	0	**2**	0
11	Can the COVID-19 vaccine cause a positive test result for the disease, such as for a PCR or antigen test?	0	0	0	**3**	**3**
12	Should I be vaccinated if I have had COVID-19?	0	0	0	**2,5**	**1,5**
13	Is the vaccine safe for children?	0	0	0,5	**2**	1
14	Do the vaccines protect against variants?	0	1,5	1	1	**2**
15	How will we know if COVID-19 vaccines are safe?	1,5	0	0	**2**	**2**

<u>Comprehension</u>

#Q	Question (comprehension)	How many times do you need to try before the VA recognized the question?					How many words are missing or transcribed wrongly?				
		Alexa	Bixby	Cortana	GA	Siri	Alexa	Bixby	Cortana	GA	Siri
1	Is there a vaccine for COVID-19?	**3**	**3**	**3**	**3**	**3**	**2**	**2**	1,5	**2**	**2**
2	When will COVID-19 vaccines be ready for distribution?	2	**3**	2	**3**	**3**	**2**	**2**	1,5	**2**	**2**
3	Will COVID-19 vaccines provide long-term protection?	**3**	**3**	**3**	**3**	2	**2**	**2**	1,5	**2**	**2**
4	How quickly could COVID-19 vaccines stop the pandemic?	2	**3**	**3**	**3**	2,5	**2**	**2**	1,5	**2**	**2**
5	What types of COVID-19 vaccines are being developed?	2,5	**3**	**3**	2,5	**3**	**2**	**2**	1,5	1,5	**2**
6	Will other vaccines help to protect me from COVID-19?	2	**3**	**3**	**3**	**3**	**2**	**2**	**2**	**2**	**2**
7	What are the benefits of getting vaccinated?	2	**3**	**3**	**3**	**3**	**2**	**2**	**2**	**2**	**2**
8	Who should get the COVID-19 vaccines?	2	**3**	2	2,5	**3**	**2**	**2**	1,5	1,5	**2**
9	Can we stop taking precautions after being vaccinated?	0,5	**3**	2,5	2	1	1	**2**	**2**	**2**	1
10	Can I have the second dose with a different vaccine than the first dose?	2	2,5	**3**	**3**	1	**2**	**2**	**2**	**2**	1
11	Can the COVID-19 vaccine cause a positive test result for the disease, such as for a PCR or antigen test?	0,5	**3**	**3**	2	**3**	1	**2**	**2**	**2**	**2**
12	Should I be vaccinated if I have had COVID-19?	2	**3**	**3**	2,5	1	**2**	1	1,5	**2**	1
13	Is the vaccine safe for children?	2	**3**	**3**	2,5	1,5	**2**	**2**	**2**	**2**	1
14	Do the vaccines protect against variants?	2	**3**	2,5	**3**	**3**	**2**	**2**	**2**	**2**	**2**
15	How will we know if COVID-19 vaccines are safe?	2,5	**3**	2,5	**3**	**3**	**2**	**2**	1,5	**2**	**2**

Relevance

#Q	Question (relevance)	Was the VA able to find an answer?					Is the VA response provided relevant to what is being asked?				
		Alexa	Bixby	Cortana	GA	Siri	Alexa	Bixby	Cortan a	GA	Siri
1	Is there a vaccine for COVID-19?	**1**	**1**	0,5	**1**	**1**	**2**	1	0,5	**2**	1
2	When will COVID-19 vaccines be ready for distribution?	0,5	**1**	**1**	**1**	0,5	0	0	0,5	**2**	0
3	Will COVID-19 vaccines provide long-term protection?	**1**	**1**	0,5	**1**	**1**	**2**	0	0	1,5	1
4	How quickly could COVID-19 vaccines stop the pandemic?	0,5	**1**	0,5	**1**	0,5	0	0	0	**2**	0
5	What types of COVID-19 vaccines are being developed?	0,5	**1**	0,5	**1**	0,5	1	1	0	**1,5**	0
6	Will other vaccines help to protect me from COVID-19?	0,5	**1**	0,5	**1**	0,5	0	0	0	**2**	0
7	What are the benefits of getting vaccinated?	0,5	**1**	0,5	**1**	**1**	0	**2**	0	**2**	1
8	Who should get the COVID-19 vaccines?	0,5	**1**	0,5	**1**	0,5	0	0	0	**0,5**	0
9	Can we stop taking precautions after being vaccinated?	0,5	**1**	0,5	**1**	0,5	0	0	0	0	0
10	Can I have the second dose with a different vaccine than the first dose?	0,5	**1**	0,5	**1**	0,5	0	0	0	**2**	0
11	Can the COVID-19 vaccine cause a positive test result for the disease, such as for a PCR or antigen test?	0,5	**1**	0,5	**1**	0,5	0	0	0	**2**	0
12	Should I be vaccinated if I have had COVID-19?	0,5	**1**	0,5	**1**	0,5	0	0	0	**2**	0
13	Is the vaccine safe for children?	0,5	**1**	**1**	**1**	0,5	0	0	1	**2**	0
14	Do the vaccines protect against variants?	0,5	**1**	**1**	0,5	**1**	1	1,5	**2**	0,5	1
15	How will we know if COVID-19 vaccines are safe?	**1**	**1**	0,5	**1**	**1**	1	0	0	**2**	1

Reability

#Q	Question (reliability)	In the VA response provided updated when compared with official answer?					Are there references citations in the VA response provided?					How credible are the reference citations?					Are there any advertisements in the VA response provided?				
		A	B	C	GA	S	A	B	C	GA	S	A	B	C	GA	S	A	B	C	GA	S
1	Is there a vaccine for COVID-19?	0	0	1	**2**	0	**1**	0	0,5	**1**	**1**	1,5	0	1	**3**	2	**1**	**1**	**1**	**1**	**1**
2	When will COVID-19 vaccines be ready for distribution?	0	0	**1**	**1**	0	0	0	0,5	**1**	**1**	0	0	0,5	**3**	**3**	**1**	**1**	**1**	**1**	**1**
3	Will COVID-19 vaccines provide long-term protection?	0	0	0	**1,5**	0,5	**1**	0	0	**1**	**1**	1,5	0	0	2	**2,5**	**1**	**1**	**1**	**1**	**1**
4	How quickly could COVID-19 vaccines stop the pandemic?	0	0	0	**1**	0	0	0	0	**1**	**1**	0	0	0	2	**3**	**1**	**1**	**1**	**1**	**1**
5	What types of COVID-19 vaccines are being developed?	0	0	0	0	0	0,5	**1**	0	**1**	**1**	0	**3**	0	**3**	2,5	**1**	**1**	**1**	**1**	**1**
6	Will other vaccines help to protect me from COVID-19?	0	0	0	**1**	0	0	0	0	**1**	**1**	0	0	0	2	**2,5**	**1**	**1**	**1**	**1**	**1**
7	What are the benefits of getting vaccinated?	0	0	0	**2**	0,5	0	**1**	0	**1**	**1**	0	**3**	0	**3**	1	**1**	**1**	**1**	**1**	**1**
8	Who should get the COVID-19 vaccines?	0	0	0	**1**	0	0	0	0	**1**	**1**	0	0	0	2,5	**3**	**1**	**1**	**1**	**1**	**1**
9	Can we stop taking precautions after being vaccinated?	0	0	0	0	0	0	0	0	0	0	0	0	0	0	0	0,5	**1**	**1**	**1**	0,5
10	Can I have the second dose with a different vaccine than the first dose?	0	0	0	**1,5**	0	0	0	0	**1**	0	0	0	0	**2**	0	**1**	**1**	**1**	**1**	0,5
11	Can the COVID-19 vaccine cause a positive test result for the disease, such as for a PCR or antigen test?	0	0	0	**2**	0	0	0	0	**1**	**1**	0	0	0	**3**	**3**	0,5	**1**	**1**	**1**	**1**

12	Should I be vaccinated if I have had COVID-19?	0	0	0	**2**	0	0	0	0	**1**	0,5	0	0	0	**2,5**	1,5	**1**	**1**	**1**	**1**	0,5
13	Is the vaccine safe for children?	0	0	1	**1,5**	0	0	0	0,5	**1**	0,5	0	0	0,5	**2**	1	**1**	**1**	**1**	**1**	0,5
14	Do the vaccines protect against variants?	0	1,5	**2**	1	1	0	**1**	**1**	0,5	**1**	0	1,5	1	1	**2**	**1**	**1**	**1**	**1**	**1**
15	How will we know if COVID-19 vaccines are safe?	0	0	0	**1**	**1**	0,5	0	0	**1**	**1**	1,5	0	0	**2**	**2**	**1**	**1**	**1**	**1**	**1**

<u>User-friendliness</u>

#Q	Question (user-friendliness)	Was the response presented in Portuguese?					Was the response presented by text and by voice?					Is the content in the VA response provided presented in a way that it can be easily understood by a lay person?				
		A	B	C	GA	S	A	B	C	GA	S	A	B	C	GA	S
1	Is there a vaccine for COVID-19?	**2**	**2**	**2**	**2**	**2**	**2**	**2**	1,5	**2**	1,5	**1**	**1**	**1**	**1**	**1**
2	When will COVID-19 vaccines be ready for distribution?	**2**	**2**	**2**	**2**	**2**	1	**2**	**2**	**2**	**2**	0,5	0	0,5	**1**	**1**
3	Will COVID-19 vaccines provide long-term protection?	**2**	**2**	**2**	**2**	**2**	**2**	**2**	1,5	1,5	1,5	**1**	0	0,5	**1**	**1**
4	How quickly could COVID-19 vaccines stop the pandemic?	**2**	**2**	**2**	**2**	**2**	1	**2**	1,5	1	**2**	0,5	0	0,5	**1**	**1**
5	What types of COVID-19 vaccines are being developed?	**2**	**2**	**2**	**2**	**2**	1,5	**2**	1,5	**2**	**2**	**1**	**1**	0,5	**1**	**1**
6	Will other vaccines help to protect me from COVID-19?	**2**	**2**	**2**	**2**	**2**	1	**2**	1,5	1	**2**	0,5	0	0,5	**1**	**1**
7	What are the benefits of getting vaccinated?	**2**	**2**	**2**	**2**	**2**	1	**2**	1,5	**2**	1,5	0,5	**1**	0,5	**1**	**1**
8	Who should get the COVID-19 vaccines?	**2**	**2**	**2**	**2**	**2**	1	**2**	1,5	**2**	**2**	0,5	0	0,5	**1**	**1**
9	Can we stop taking precautions after being vaccinated?	1	**2**	**2**	**2**	1	0,5	**2**	1,5	**2**	1	0	0	**0,5**	0	0
10	Can I have the second dose with a different vaccine than the first dose?	**2**	**2**	**2**	**2**	1	1	**2**	1,5	1	1	0,5	0	0,5	**1**	0
11	Can the COVID-19 vaccine cause a positive test result for the disease, such as for a PCR or antigen test?	1	**2**	**2**	**2**	**2**	0,5	**2**	1,5	1,5	**2**	0	0	0,5	**1**	**1**
12	Should I be vaccinated if I have had COVID-19?	**2**	**2**	**2**	**2**	1	1	**2**	1,5	**2**	1	0,5	0	0,5	**1**	0,5
13	Is the vaccine safe for children?	**2**	**2**	**2**	**2**	1	1	**2**	**2**	1,5	1	0,5	0	0,5	**1**	0,5
14	Do the vaccines protect against variants?	**2**	**2**	**2**	**2**	**2**	1	1,5	**2**	**2**	1,5	**1**	**1**	**1**	0,5	**1**
15	How will we know if COVID-19 vaccines are safe?	**2**	**2**	**2**	**2**	**2**	**2**	**2**	1,5	1,5	1,5	0,5	0	0,5	**1**	**1**
